# Optimization of environmental air sampling for viral metagenomics in a cave-roosting bat assemblage

**DOI:** 10.1186/s42522-026-00218-3

**Published:** 2026-05-16

**Authors:** Caleb A. Huntington, Cassandra M. Bonavita, Heather L. Wells, Jay D. Tiemann, Isamara Navarrete-Macias, Reed F. Johnson, Lisa E. Hensley, Simon J. Anthony

**Affiliations:** 1 Department of Pathology, Microbiology, and Immunology, School of Veterinary Medicine, University of California - Davis, Davis, CA USA; 2 Zoonotic and Emerging Disease Research Unit, National Bio and Agro-Defense Facility, Agricultural Research Service, United States Department of Agriculture, Manhattan, KS USA

**Keywords:** Environmental air sampling, Bats, Coronaviruses, Biosurveillance, Wildlife

## Abstract

**Background:**

Environmental air sampling holds significant potential as a tool for viral surveillance. Its use in agricultural and indoor settings has demonstrated its feasibility and effectiveness but despite this, it has rarely been used in wildlife settings.

**Methods:**

To enable future applications, we optimized key parameters in air sampling methodology using a cave-roosting bat assemblage as a model system. We systematically investigated the impact of sampling conditions (flow rate, sampling duration, and sampling location/deployment time) and post-sampling treatments (DNA/RNA Shield ratios and secondary filtration) on three viral metrics – total mammalian virus abundance, mammalian RNA virus abundance, and Shannon diversity index – generated from next-generation sequencing data.

**Results:**

We first showed that air sampling can recover broad viral diversity, including alphacoronaviruses and betacoronaviruses. The sampling conditions for maximizing viral metrics were larger air sample volumes (≥24,000 liters) and sampling inside the cave while the bats were roosting, as opposed to at the cave entrance during emergence. Post-sampling treatments had limited impact on viral metrics, but their application may vary depending on the objectives of the study.

**Conclusion:**

This work provides a proof-of-concept for applying air sampling for wildlife viral surveillance in a cave-roosting bat assemblage and identifies key sampling parameters.

**Supplementary Information:**

The online version contains supplementary material available at 10.1186/s42522-026-00218-3.

## Introduction

Environmental air sampling has a long history as a tool for virus detection. It was first applied in the 1940s when DeLay and colleagues recovered exotic Newcastle disease virus (NDV) from air in poultry houses [1]. In that study, air was drawn from pens containing both vaccinated and unvaccinated chickens during a natural outbreak, and infectious virus was successfully isolated in embryonated eggs - providing the first demonstration that naturally infected animal viruses could be recovered directly from air. Since then, the approach has been used for the detection of other livestock pathogens such as foot-and-mouth disease virus (FMDV) [2–4], porcine circovirus (PCV) [5–7], and porcine reproductive and respiratory syndrome virus (PRRSV) [8, 9], as well as zoonotic viruses in agricultural settings and live-animal markets including avian influenza A virus (IAV) [10–15], Middle East respiratory syndrome coronavirus (MERS-CoV) [16], and severe acute respiratory syndrome coronavirus-2 (SARS-CoV-2) [17–19].

In contrast, applications of air sampling in wildlife settings are much less common. The earliest example dates to 1968, when rabies virus was isolated from the air of bat cave in Texas [20]. More recently, IAVs (subtypes: H5, H7, and H9) were detected molecularly in air samples collected from winter, wetland habitats of migratory birds in Taiwan [21]. Beyond these isolated cases, no other study has systematically evaluated how air sampling performs in wildlife contexts, or which parameters influence viral detection. This gap is notable given the recognized role of wildlife as reservoirs for zoonotic viruses [22]. Moreover, even in agricultural and human settings, prior studies have often neglected to report critical sampling details such as flow rate, sample volume, and sampling location, limiting comparability across studies and obscuring the factors that drive successful virus detection [23–25]. Establishing methodological baselines in wildlife systems is needed to improve surveillance for emerging pathogens.

To address this gap, we designed a study to optimize environmental air sampling for wildlife viral surveillance. We focused on methodological parameters that prior studies highlighted as potentially important but have not yet been tested in a systematic way. Specifically, we evaluated two categories of parameters – sampling conditions (i.e., factors during air collection) and post-sampling treatments (i.e., steps applied after collection) – and assessed their influence on viral detection using next-generation sequencing (NGS). As a model system, we selected cave-roosting bats. Certain bat species have been identified as reservoirs for high-impact zoonoses including being strongly associated with coronaviruses, making them a priority for viral surveillance [26, 27]. In addition, some bat species roost in large, dense colonies in enclosed environments [28] where aerosolized viruses are likely to accumulate and persist, providing ideal conditions to evaluate how sampling parameters influence viral detection.

For sampling conditions, we investigated three parameters that could influence detection: flow rate, sampling duration, and sampling location/deployment time. Higher flow rates in dry-filter samplers can accelerate desiccation on the filter, increasing mechanical stress on collected particles and potentially reducing nucleic acid recovery for sequencing [29]. Longer sampling durations increase the total volume of air collected and may therefore improve viral detection, unless the airborne viral community is well-mixed and smaller volumes already provide a representative sample [23]. Finally, sampling location and deployment time are also likely to be critical. Sampling outside the cave entrance would be logistically easier and reduce the need to enter the cave, whereas sampling inside the cave places the sampler in closer proximity to the bats and may increase the likelihood of detecting viral nucleic acids [30–32]. Likewise, deploying samplers while bats are roosting versus during emergence may influence detection, as host movement and air mixing increase during periods of activity. We hypothesized that lower flow rates, longer sampling durations, and sampling inside (versus outside) the cave would improve the measured viral metrics.

Beyond collection itself, sample preservation and processing are also critical, particularly in wildlife settings where immediate access to laboratory facilities is often limited. One key consideration is the use of lysis buffers that stabilize nucleic acids at ambient temperature; however, manufacturer guidelines for sample-to-buffer ratios (e.g., for DNA/RNA Shield) have not been established for air samples. Another point of uncertainty is whether the eluate from washed filters should be passed through a secondary filter to remove abundant non-viral material (e.g., bacteria, fungi) that can dominate sequencing libraries [33]. We hypothesized that DNA/RNA Shield ratios would be insignificant while secondary filtering through a 0.45 µM filter would improve viral metrics.

By evaluating both sampling conditions and post-sampling treatments, this study provides a framework for methodological optimization and demonstrates its utility as a practical tool for wildlife viral surveillance. Our goal was not to prescribe a single “best” approach, but rather to test whether commonly recommended parameters truly influence viral detection. The most appropriate choices will likely depend on the specific objectives of future studies; for example, whether the priority is maximizing viral diversity, recovering intact genomes, or reducing non-viral background. Our results therefore provide practical information on how different variables influence viral detection, offering guidance for the design of future wildlife surveillance studies.

**Fig. 1:**
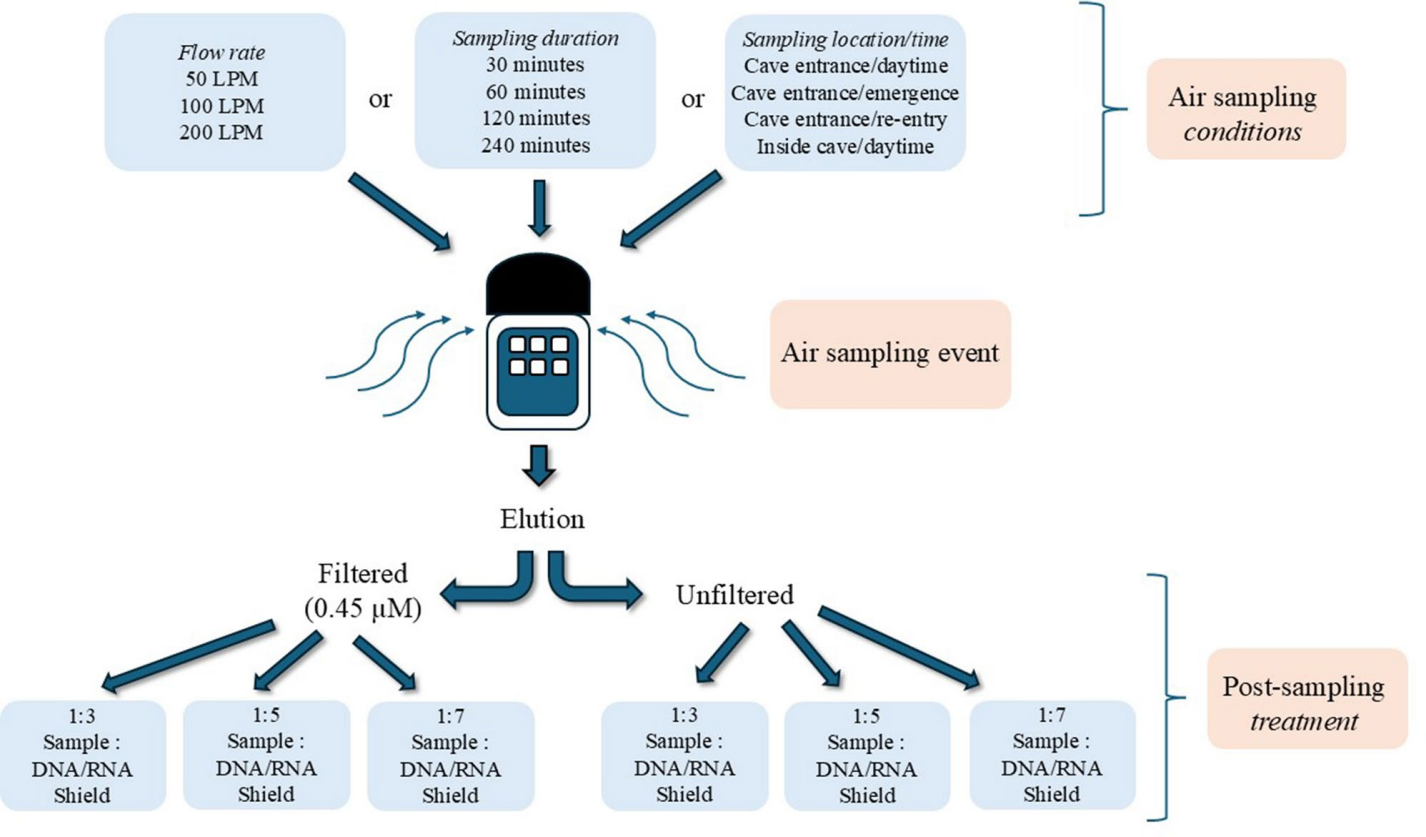
Diagram illustrating air sampling condition experiments and how samples were split for a subset of air sampling events. Depending on the experiment, one of the following air sampling conditions was varied between air sampling events: flow rates, sampling durations, or sampling locations/deployment times. Following filter elution, the resulting sample was divided in half for secondary filtering through a 0.45 µM filter or left unfiltered. Then, each half was split into three and preserved in different ratios of sample to DNA/RNA Shield

## Methods

### Field site

This study was conducted at Culebrones Cave in the Mata de Plátano Natural Reserve, Puerto Rico. Culebrones Cave is a karstic hot cave with six bat species (*Brachyphylla cavernarum*, *Erophylla bombifrons*, *Monophyllus redmani*, *Mormoops blainvillii*, *Pteronotus portoricensis*, and *Pteronotus quadridens*) that roost there year-round. A recent study estimates the assemblage varies seasonally from 76,028 (March 2021) to 158,300 (October 2020) total individuals [34]. Field work was done in July of 2023 and July and August of 2024. During this time period, previous work has shown that some of the species are likely to be pregnant and/or lactating [35]. Since reproductive status has been shown to have an effect on viral shedding [36], we limited our experimental windows to short intervals (1–3 weeks) to minimize the effect of viral shedding dynamics.

Temperature and relative humidity were measured using the Kestrel 3000 weather meter (Kestrel Instruments, SKU: 0830) for each air sampling event.

*Air sampling conditions* Air samples were collected using the AirPrep Cub Sampler (Innovaprep, Drexel, MO, USA, Catalog number ACD210). The AirPrep Cub was handled wearing nitrile gloves and N95s and cleaned with Lysol wipes before and after each air sampling event. For air sampling events inside the cave, the AirPrep Cub was mounted on a tripod approximately 1 m off the ground, just past the entrance of the largest hot chamber in the cave (maximum height of this chamber is approximately 40 m). For air sampling events at the cave entrance, the AirPrep Cub was placed directly on the ground just inside the cave entrance on a ledge on the downward slope leading into the cave (Fig. S1). To assess how collection parameters influenced viral detection, we systematically varied flow rate, sampling duration, and sampling location and deployment time (sampling details in Table S1). To evaluate the effect of flow rate, samples were collected at 50, 100, and 200 L per minute (LPM) while keeping the total air volume fixed at 6000 L, corresponding to sampling durations of 120, 60, and 30 min respectively. These collections were conducted over one week in triplicate (per condition) both inside the cave while bats were roosting (daytime, sampling started between 11:00 and 15:00) and at the cave entrance during emergence (evening, sampling started between 17:45 and 22:30). To evaluate the effect of increasing sample volume from increasing *sampling duration*, air sampling was conducted for 30, 60, 120, and 240 min using a fixed flow rate of 100 LPM. These were collected over a two-week period in triplicate at the same two locations and times. To evaluate the effect of increasing sample volume from increasing *flow rates*, air sampling was conducted at 50, 100, and 200 LPM with a fixed sampling duration of 60 min. These samples were collected over a 1.5-week period and collected in triplicate (this was only done inside the cave). To evaluate the effect of location and deployment time, samples were collected during behaviorally relevant times: inside the cave while bats were roosting, the cave entrance during the daytime when no bats were present, the cave entrance during evening emergence, and the cave entrance during morning re-entry. These collections were conducted over three weeks (*n* = 5 per condition) at a flow rate of 200 LPM for 180 min. Additional sampling events that were not part of the experiments on sampling conditions were conducted throughout the summer and included in evaluation of post-sampling treatments.

*Post-sampling treatments* Following collection, air sampling filters were eluted in the field using the Tris elution kit (Innovaprep, Drexel, MO, USA; Catalog number AC08100T). Each eluate was split, with half passed through a 0.45 µM syringe filter and the other half left unfiltered. To evaluate the effect of nucleic acid stabilization, eluates were preserved in DNA/RNA Shield (Zymo Research, Irvine, CA, USA; R1200-125) at three different sample-to-buffer ratios (1:3, 1:5, 1:7; Fig. 1). All samples were kept for one to eleven days at 4 °C (well within manufacturer recommendations) and then shipped to UC Davis at ambient temperature and stored at -80 °C until extraction. For a negative control, an air sampling filter was eluted in the laboratory at UC Davis.

*Laboratory processing* Total nucleic acids were extracted from each sample using Quick-DNA/RNA Viral Kit (Zymo Research, Irvine, CA, USA; D7020) and eluted using 75 µL of RNase-free H_2_O. Given the different ratios of sample to DNA/RNA Shield, different sample volumes for the different ratios were extracted so that they corresponded to 100 µL of eluate (e.g. 400 µL for an air sample stored in a 1:3 sample-to-buffer ratio). For next-generation sequencing (NGS), library preparation was performed using the *Twist Total Nucleic Acids Library Preparation EF Kit 2.0 for Viral Pathogen Detection and Characterization*. Samples were then sequenced to a depth of approximately 100 million 150-bp paired-end reads using Illumina Novaseq X.

*Bioinformatics pipeline* The resulting NGS fastq files for each sample were preprocessed for adapter removal, trimming, and quality filtering (fastp, v0.22.0, < Q20) [37]. Reads mapping to the human genome (GCF_000001405.40) were subtracted (bowtie2, v2.5.4) [38] before *de novo* assembly (megahit v1.2.9, –meta-large) [39]. A custom diamond (v.2.1.9) [40] database was constructed using viral protein sequences from UniProtKB (all sequences under taxid 10239, downloaded on 2024-10-07). For taxonomic classification, accession numbers were mapped to taxids and the NCBI nodes.dmp and names.dmp files were incorporated into the database. Contigs greater than 500 bp were run using diamond blastx (--evalue 1E-03, --max-target-seqs 1, --very-sensitive, --block-size 3.0, --query-cover 10) against the custom viral database. Viral family and genus were added to the blastx output using NCBI lineages (2024-06-13). Results were then filtered to only include mammalian viral families (list modified from ViralZone: *Adenoviridae*,* Anelloviridae*,* Arenaviridae*,* Arteriviridae*,* Asfarviridae*,* Astroviridae*,* Bornaviridae*,* Caliciviridae*,* Circoviridae*,* Coronaviridae*,* Filoviridae*,* Flaviviridae*,* Hantaviridae*,* Hepadnaviridae*,* Hepeviridae*,* Herpesviridae*,* Kolmioviridae*,* Nairoviridae*,* Orthoherpesviridae*,* Orthomyxoviridae*,* Papillomaviridae*,* Paramyxoviridae*,* Parvoviridae*,* Peribunyaviridae*,* Phenuiviridae*,* Picornaviridae*,* Pneumoviridae*,* Polyomaviridae*,* Poxviridae*,* Reoviridae*,* Retroviridae*,* Rhabdoviridae*, and *Togaviridae*). Contigs were then rerun using diamond blastx (--evalue 1E-03, --max-target-seqs 1, --sensitive, --block-size 5.0, --query-cover 10) against a diamond database built from the NCBI nr database (downloaded on 2024-02-07). Any non-viral hits were removed. Deduplicated reads were mapped back to identified contigs using coverM (v0.7.0, -m count) [41]. To ensure that these contigs were likely of bat origin, every contig was manually assessed using the top blastn hits from the nt database to then remove matches that were clearly insect (e.g., *Periplaneta fuliginosa* densovirus) or human (e.g., human papillomavirus).

To classify all air sampling reads to high taxonomic orders, Kraken2 (v2.1.3) [42] with the core_nt database (2024-06-26) was used. Kraken2 output was modified by ignoring reads that were classified as SARS-CoV-2 as there was evidence that Kraken2 was misidentifying reads as SARS-CoV-2 which inflated the proportion of reads classified as viral. This was confirmed by mapping air sample NGS reads to the reference sequence of SARS-CoV-2 (NC_045512) to verify there was not widespread contamination.

### Viral detection measures

Viral detection was quantified using three outcomes. Total mammalian virus abundance was measured as the sum of deduplicated reads mapping to contigs classified as mammalian viral families (in reads per million, RPM). Mammalian RNA virus abundance was calculated in the same way but restricted to contigs classified as RNA virus families. The Shannon diversity index was calculated at the viral genus level using average read depth per contig as a measure of abundance to account for differences in contig length.

### Statistics

For sampling conditions, to evaluate how flow rate and sampling duration influenced the viral detection outcomes described above, we used LOESS regressions (span = 0.75) with significance evaluated using Spearman’s rank correlation. Categorical comparisons for sampling location and deployment time were evaluated using the Kruskal-Wallis test with Dunn’s post-hoc test. For modeling sampling conditions on specific viral families, a logistic regression was employed. To test how post-sampling treatments influenced viral detection outcomes, we used generalized linear mixed models (GLMMs). All GLMMs were fit using a Tweedie distribution with log link using the glmmTMB package [43]. Zero-inflated models were also considered but provided a poorer fit based on simulated residuals from dHARMA [44]. Effect size plots were generated from the model outputs using ggplot2 [45].

## Results

**Fig. 2:**
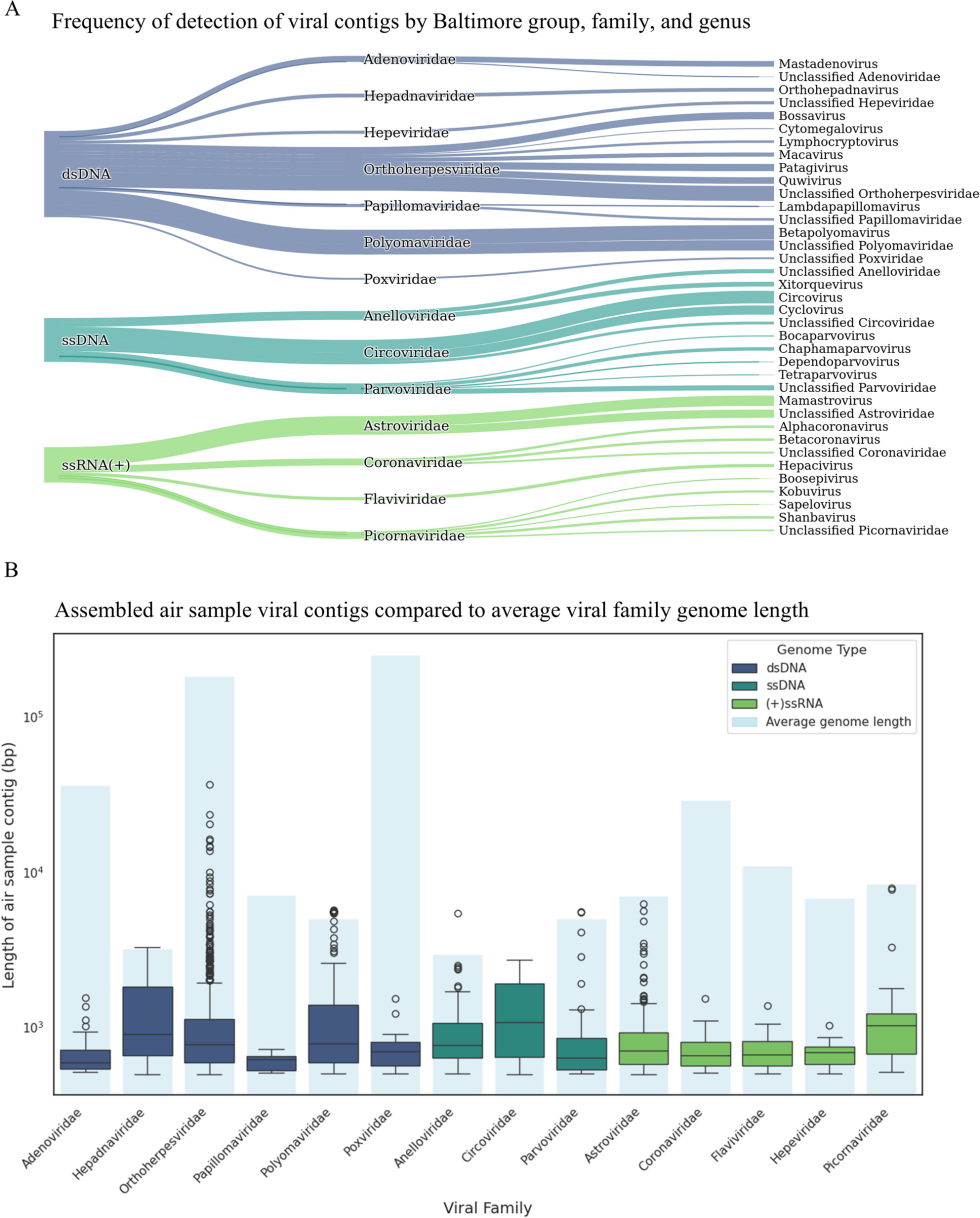
Air sampling broadly detects mammalian viral families. (**A**) Alluvial plot summarizing the detected Baltimore groups, families, and genera across all air sampling events. The width of the flow to the genus node corresponds to detection rate for that viral genus. (**B**) Box plot (logarithmic scale) illustrating the viral contig lengths by viral family across air sampling events. Blue bars in the background represent the average length of a viral genome in that viral family according to ICTV

### Air sampling detects broad mammalian viral diversity

We conducted a total of 115 air sampling events in July 2023 and July-August 2024. For a subset of air sampling events, the eluate was split in half for evaluating secondary filtration with a subset of samples then split into three for evaluating sample: DNA/RNA Shield ratios for a total of 273 samples (Fig. 1). Of the 100 million reads generated for each sample from NGS, on average, 2.44% ± 6.41% were viral based on taxonomic binning of reads by Kraken 2 (Fig. S2). Following *de novo* assembly, we excluded contigs whose best matches were non-mammalian viruses (e.g., insect- or plant-associated) and retained putative mammalian viral contigs for downstream analysis. Across all sampling conditions, contigs were classified to 14 mammalian viral families comprising 25 viral genera (Fig. 2A). The most frequently represented were contigs assigned to the *Orthoherpesviridae* (including both gamma- and betaherpesviruses; 52.2% of sampling events), followed by *Polyomaviridae* (42.6%) and *Circoviridae* (37.4%). Contigs assigned to *Astroviridae* were the fourth most common (35.7%) and the most frequently detected RNA viruses. We also identified contigs classified as alphacoronaviruses and betacoronaviruses, with *Coronaviridae* represented overall in 15.6% of sampling events. Following this approach, no contigs were classified as viral in the negative control.

**Fig. 3:**
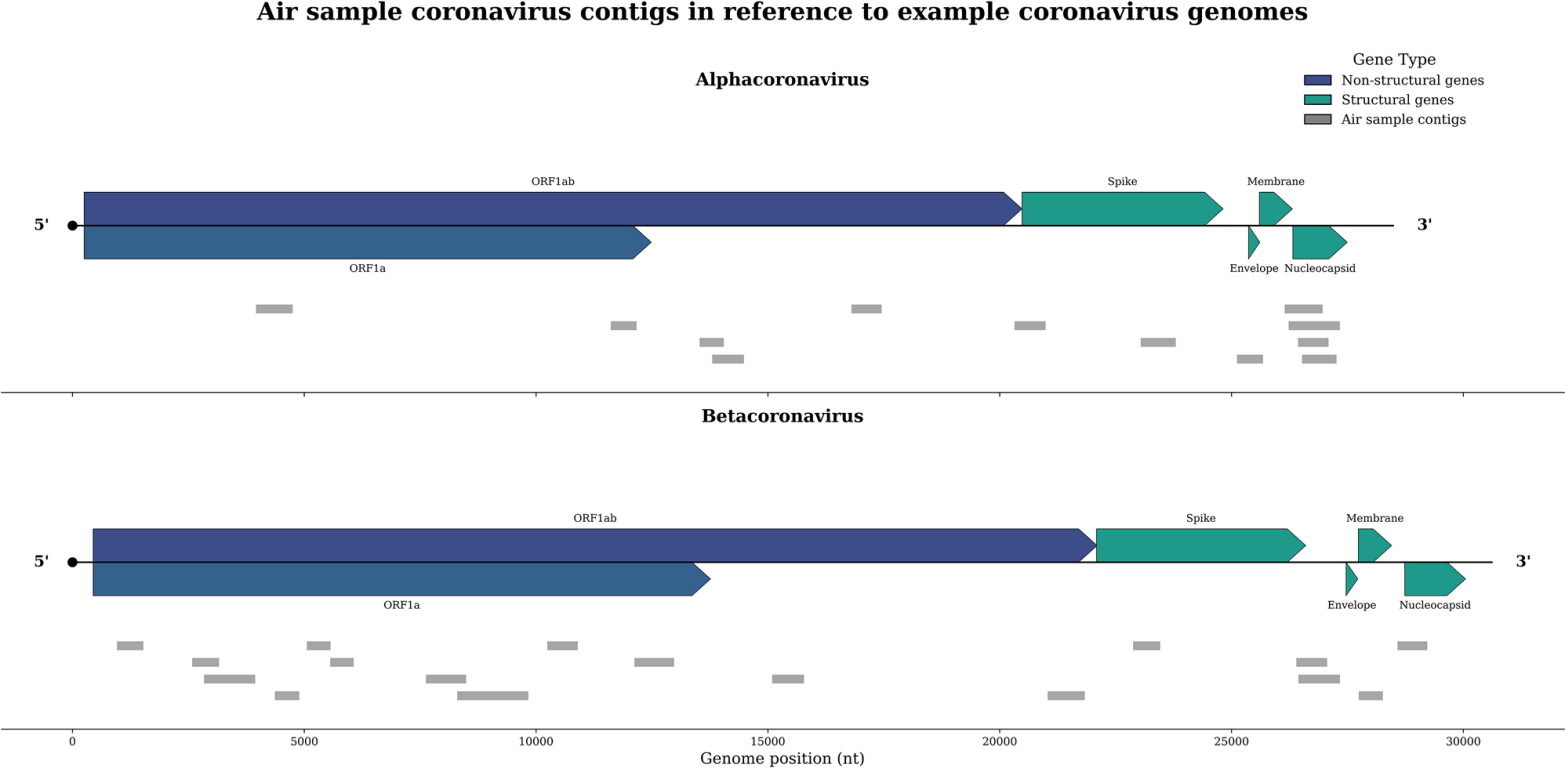
Coronavirus contigs aggregated from all air sampling events (gray bars) were approximately aligned with example alphacoronavirus and betacoronavirus genomes with their placement determined by the genomic region of their top BLASTn or BLASTx hit

Out of the fourteen viral families detected, air sampling produced contigs approaching full-length viral genomes for seven of the families (dsDNA: *Hepadnaviridae*,* Polyomaviridae*; ssDNA: *Anelloviridae*,* Circoviridae*,* Parvoviridae*; (+)ssRNA: *Astroviridae*,* Picornaviridae*) (Fig. 2B). For the remaining viral families, contigs were shorter but still sufficient for classification at the genus level (summary in Table S2). Within *Coronaviridae*, only short contigs were assembled, with no apparent bias towards particular genomic regions (Fig. 3). When aggregated across sampling events, these coronavirus contigs spanned the genome, indicating broad but fragmented coverage.

### Sampling conditions drive viral detections

To assess the impact of sampling conditions, we systematically varied flow rate, sampling duration, and sampling location and time restricted to short time periods (1–3 weeks). Restricting experiments to these windows minimized potential confounding from natural fluctuations in viral shedding. Viral detection was evaluated using three outcomes: total mammalian virus abundance, mammalian RNA virus abundance, and the Shannon diversity index.

**Fig. 4:**
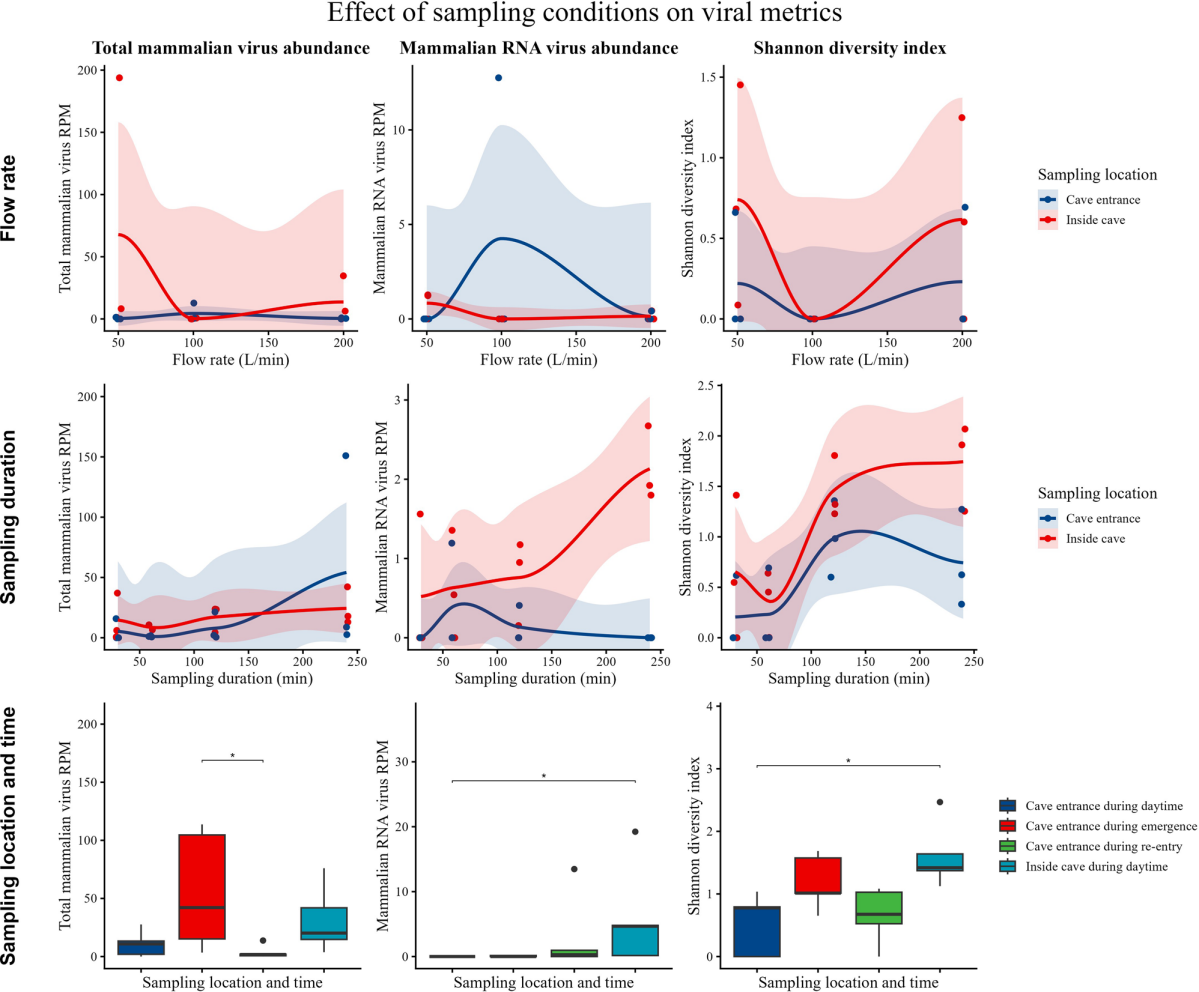
Investigating the effect of air sampling conditions on three viral metrics. The top row plots LOESS regressions (span = 0.75) comparing flow rate with total mammalian virus abundance (left), mammalian RNA virus abundance (middle), and Shannon diversity index (right). Experiments were conducted both inside the cave while the bats were roosting and at the cave entrance during emergence. Sampling duration was adjusted inversely to maintain a constant air sample volume of 6000 L (per group and condition, *n* = 3 air sampling events). Significance was assessed using Spearman’s rank correlation. The middle row plots LOESS regressions for sampling duration against the same viral metrics with a fixed flow rate of 100 LPM (per group and condition, *n* = 3 air sampling events). Significance was again evaluated with Spearman’s rank correlation. For visualization only, one outlier was removed from the plot of total mammalian viral abundance but the outlier was retained in statistical analyses. The bottom row plots sampling location and deployment time (inside the cave during the daytime, the cave entrance during the daytime, the cave entrance during emergence, and the cave entrance during re-entry) against the three viral metrics (per group and condition, *n* = 5 air sampling events). Each air sampling event was done under a 200 LPM flow rate for 180 min. Significance was evaluated using Kruskal-Wallis tests with Dunn’s post-hoc test. For all analyses, samples did not undergo secondary filtration and were stored in a 1:3 ratio of sample to DNA/RNA Shield

When flow rate was varied (50, 100, 200 LPM) while holding sample volume constant at 6000 L (sampling durations adjusted inversely), no significant effect was observed for any of these outcomes at either location (Fig. 4, top row; significance Table S3). In contrast, varying sampling duration (30, 60, 120, and 240 min) at a fixed flow rate of 100 LPM, corresponding to larger air volumes, produced significant positive effects inside the cave for mammalian RNA virus abundance and Shannon diversity (Fig. 4, middle row, significance Table S3). Mammalian RNA virus abundance increased sharply between the 120- and 240-minutes sampling durations, while the Shannon diversity began to level off. At a flow rate of 100 LPM, the 240 min of air sampling corresponds to a total sample volume of 24,000 L. Finally, when sampling duration was held constant while varying flow rates (as another means of varying sample volume), Shannon diversity was positively correlated with increased flow rates (significance Table S3). Together, these results indicate that larger sample volumes, driven either by longer sampling durations or higher flow rates, are critical for viral detection.

Sampling location and deployment time also had strong effects. When flow rate and sampling duration were held constant (200 LPM for 240 min), samples collected inside the cave during the daytime yielded significantly higher mammalian RNA virus abundance and Shannon diversity than samples collected at the cave entrance during the daytime (Fig. 4, bottom row; descriptive statistics Table S4; significance testing, Table S5). Notably, air sampling at the entrance in the absence of bats still detected mammalian viruses (primarily contigs classified to *Polyomaviridae*). Temperature and humidity were recorded for each air sampling event and found to be stable within sampling locations and deployment times (emergence: relative humidity 85–100%, temperature 25–28 °C; inside the cave: relative humidity 80–90%, temperature 31–32 °C).

To further evaluate location effects, we compared detection rates by viral family across all sampling events. Several families — *Anelloviridae*, *Coronaviridae*, *Flaviviridae*, and *Hepadnaviridae* — were only represented in samples collected inside the cave. For other families, the majority of detections occurred inside the cave, including *Papillomaviridae (91%)*,* Circoviridae* (89%), *Astroviridae* (87%), *Poxviridae* (83%), *Hepeviridae* (82%), *Picornaviridae* (82%), and *Parvoviridae* (81%). Finally, all viral families observed at the cave entrance were also observed inside the cave but not vice-versa, suggesting that air sampling inside the cave captures the broadest representation of the viral diversity present.

### Post-sampling treatments have limited impact

To assess the impact of post-sampling treatments, we tested whether DNA/RNA Shield ratios or secondary filtration altered viral detection. These experiments allowed us to evaluate whether preservation and processing steps introduced systematic biases. Viral detection was evaluated using the same three outcomes as above: total mammalian virus abundance, mammalian RNA virus abundance, and Shannon diversity.

**Fig. 5:**
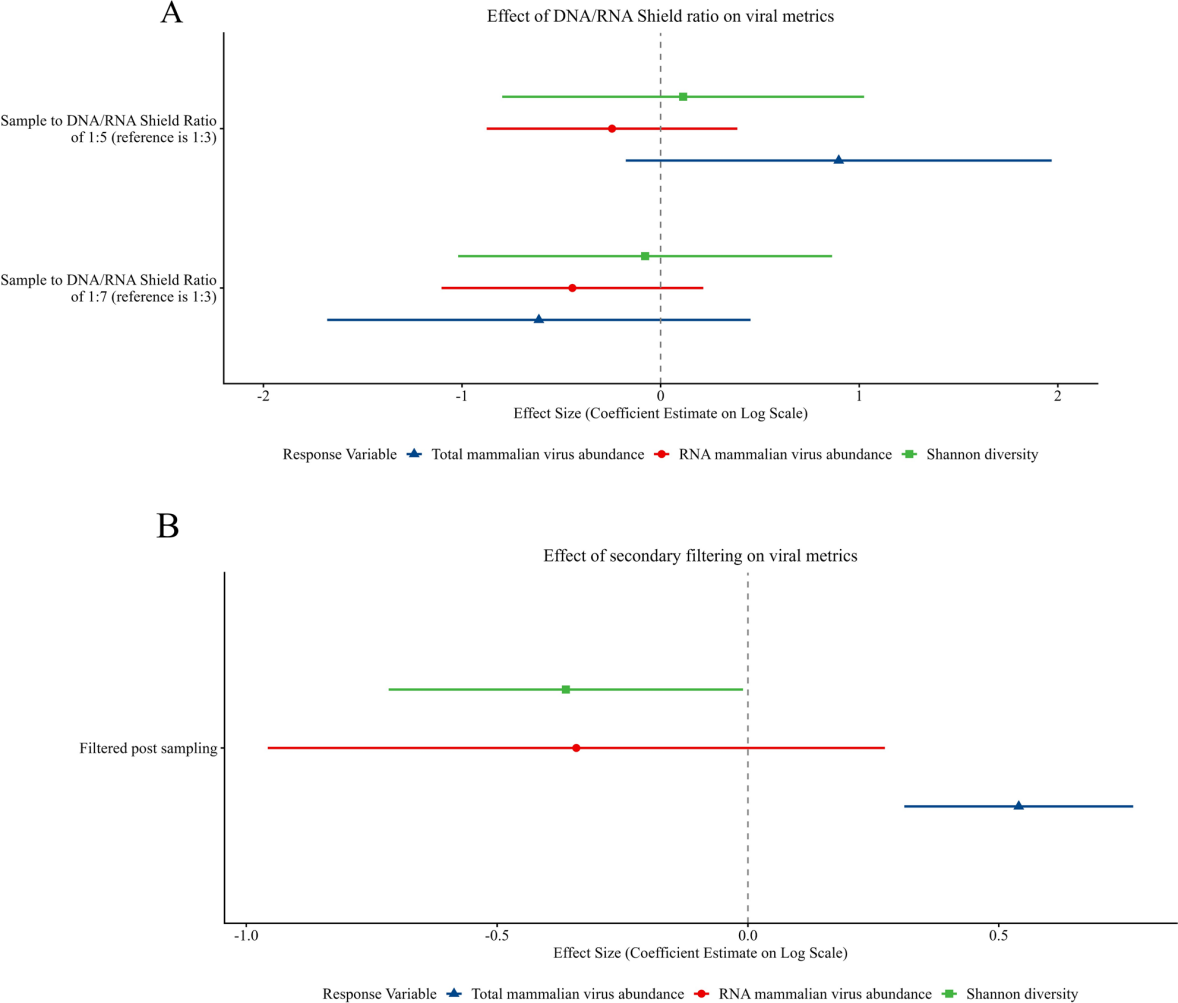
Effects of post-sample treatment on viral metrics estimated by generalized linear mixed models (GLMMs). Models assessed three response variables: total mammalian virus abundance, mammalian RNA virus abundance, and the Shannon diversity index. The models testing sample to DNA/RNA Shield ratios (**A**, *n* = 25) and secondary filtering (**B**, *n* = 90) only included the predictor of interest and air sampling event as a random effect. Effect sizes are expressed as the multiplicative change relative to the reference category (**A**: sample to DNA/RNA Shield ratio of 1:3, **B** unfiltered post-sampling)

For DNA/RNA Shield ratios, GLMMs showed no significant differences among the three ratios tested (1:3, 1:5, 1:7) for any viral metric (Fig. 5A). This indicates that, with respect to nucleic acid preservation for sequencing, all three ratios performed similarly. For secondary filtration, the GLMMs had varying results compared to unfiltered samples (Fig. 5B). We observed that filtration increased total viral abundance (β = 0.54 ± 0.12 SE, *p* = 3.5E-06) corresponding to 72% more reads per million, but reduced Shannon diversity (β = -0.36 ± 0.18 SE, *p* = 0.044) corresponding to a 30% decrease (Table S6). Logistic regression model results (including sample volume, location, and filtration as predictors) for specific viral families based on detection rates are provided in Table S7. From these models, secondary filtering significantly decreased the number of reads for Astroviridae (OR = 0.19, 95% CI: 0.067–0.49, p_BH_ = 0.0045) and Orthoherpesviridae (OR = 0.45, 95% CI: 0.24–0.84, p_BH_ = 0.036). The number of reads for Papillomaviridae was increased but was found to be non-significant. When viewed alongside the viral metrics used for assessing secondary filtering, the observation is consistent with a process that enriches some viral families while excluding others.

We also considered whether storage time at 4 °C resulted in sample degradation using similar GLMMs with viral metrics as response variables, the sampling conditions and treatments that were identified as significant as predictors (sample volume, sampling location, sampling duration, and secondary filtering), and sampling week as a random effect. We found no evidence of degradation of viruses as time at 4 °C increased (Table S8).

## Discussion

Environmental air sampling successfully detected a wide range of mammalian viruses in a bat cave in Puerto Rico, highlighting the value of this approach for surveillance of densely aggregated wildlife populations. The detection of contigs within *Coronaviridae* is of particular interest as bats are associated with, or the hosts of an evolutionary ancestor of, at least three major coronavirus spillover events (SARS-CoV, MERS-CoV, and SARS-CoV-2) [46–48], and extends prior work utilizing air sampling for the detection of coronaviruses of animal origin in farms [49], animal markets [15], and experimentally infected settings [50]. Overall, our study shows that sampling conditions were more important than post-sampling treatments for viral detection across multiple metrics. Sampling higher volumes of air, driven either by longer sampling durations or higher flow rates, had a significant, positive effect on viral metrics as did sampling inside the cave. By optimizing sample collection – collecting ≥ 24,000 L of air from inside the cave – viral detection was substantially improved. For example, coronaviruses were classified in 35.0% of optimized events compared with 11.7% of non-optimized events.

In contrast, post-sampling treatments had limited influence on sequencing outcomes. We observed that DNA/RNA Shield ratios performed equivalently for nucleic acid preservation. In addition, secondary filtration increased total viral abundance but reduced diversity, an effect likely moderated here by deep sequencing (~ 100 million reads per sample) but potentially important in studies with shallower sequencing or when targeting specific viral families. Together, these findings emphasize that optimizing collection volume and location is the most reliable strategy for maximizing viral detection in this system, while the value of specific post-sampling treatments will depend on study goals.

Our study highlighted certain limitations of air sampling as a biosurveillance tool. Although many complete or near-complete viral genomes were recovered from other families, coronavirus sequences were only assembled as short non-overlapping contigs drawn from different air sampling events. This prevents resolution to taxonomic ranks lower than genus (e.g., species, strain, or viral operational taxonomic unit.), and phylogenetic placement based on such fragments would be unreliable, particularly for a viral family where recombination is common [51]. For example, two contigs from opposite ends of the genome could align to different parental viruses even if they both belong to the same recombinant strain. Importantly, this pattern does not necessarily indicate that coronaviruses are inherently less detectable by air sampling; rather, it likely reflects the relatively low abundance of coronavirus material in the sampled air at that time. Furthermore, even for viral families with near-complete genomes, the short-read, *de novo* assembly approach could result in chimeric assemblies of closely related viruses. One alternative for future studies would be to explore long-read sequencing to bypass this issue. Additionally, more sensitive laboratory approaches, such as enrichment prior to sequencing, could increase recovery of longer contigs. However, enrichment carries trade-offs, including potential loss of more divergent viruses or other non-viral material (e.g., bacteria, fungi, host eDNA) that may also be of interest.

We also note the absence of mammalian (-)ssRNA viruses in our data. This result is notable because certain bat species are established hosts of many such viruses, including members of the family *Paramyxoviridae* [52–54]. It remains unclear whether this absence reflects traits that affect their detectability in air samples (e.g., environmental stability) or simply that these viruses are not present in this bat assemblage, at least at the time of sampling. Rabies virus, another (-)ssRNA virus expected in North American bat communities, was also not detected [55, 56]. While rabies cases have been documented in Puerto Rico in dogs and humans, there is only limited serological evidence that the virus is present in bats in Puerto Rico and it has yet to be detected molecularly or isolated [57]. Future studies should include concurrent individual bat sampling coupled with longitudinal air sampling to better assess whether the absence of (–)ssRNA viruses reflects biological traits of these viruses or the ecological context of the bat community, and to determine the extent to which air sampling can recapitulate host viral diversity.

## Conclusion

In this study, we demonstrate the feasibility of environmental air sampling as an effective and scalable approach for enhancing wildlife viral surveillance. Air sampling broadly detects mammalian viral families, including coronaviruses, and we show that sampling conditions and treatments can be optimized to improve viral metrics. We found that air sample volumes of at least 24,000 L and sampling inside the cave were the most important sampling conditions for viral detection. Secondary filtering through a 0.45 µM filter decreased measured viral diversity but increased overall viral reads and the tested ratios of sample to DNA/RNA Shield were not significantly different from one another. Environmental air sampling represents a promising tool for non-invasive biosurveillance.

## Supplementary Information

Below is the link to the electronic supplementary material.

Supplementary Material 1

Supplementary Material 2

## Data Availability

The next-generation sequencing datasets generated and analyzed during the current study are available in the Sequence Read Archive (SRA) under the BioProject: PRJNA1419286.

## Abbreviations

DNA: Deoxyribonucleic acid
eDNA: Environmental DNA
FMDV: Foot-and-mouth disease virus
GLMM: Generalized linear mixed model
IAV: Influenza A virus
LPM: Liters per minute
MERS-CoV: Middle East respiratory syndrome coronavirus
NDV: Newcastle disease virus
NGS: Next-generation sequencing
PCV: Pporcine circovirus
PRRSV: Porcine reproductive and respiratory syndrome virus
RNA: Ribonucleic acid
RPM: Reads per million
SARS-CoV-2: Severe acute respiratory syndrome coronavirus-2
ssRNA: Single-stranded RNA
