# Supplementary material for: Optimization of environmental air sampling for viral metagenomics in a cave-roosting bat assemblage
Source: One Health Outlook. 2026 May 16;8:34. doi: 10.1186/s42522-026-00218-3 (PMC13352922; doi:10.1186/s42522-026-00218-3)
Supplement: Supplementary file 2 — Supplementary Material 2 [file 42522_2026_218_MOESM2_ESM.docx]

**Supplemental Information:**

**
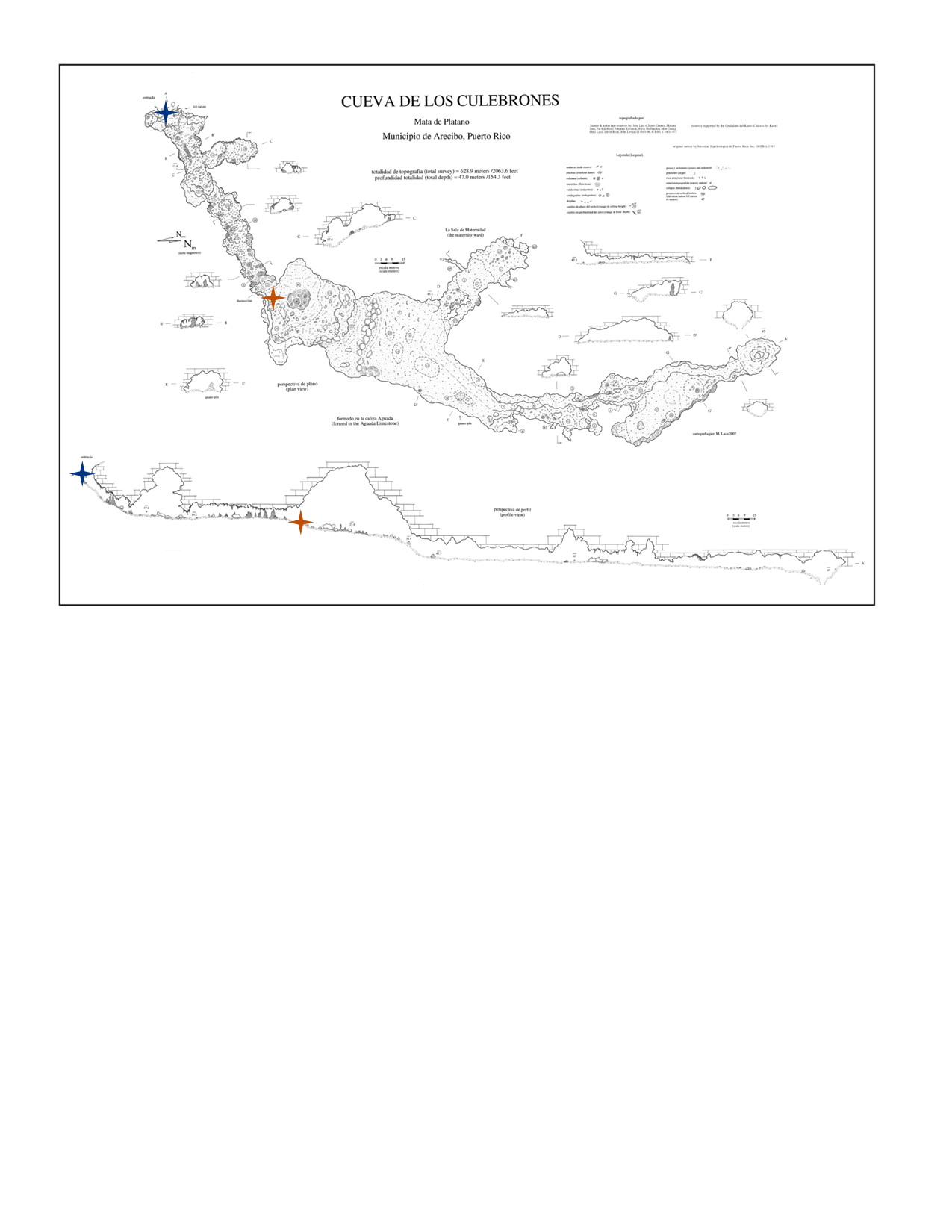
**

Supplemental Figure 1: Map of Cueva de los Culebrones (Culebrones Cave), Mata de Plátano Natural Reserve, Puerto Rico. This cave was first surveyed by La Sociedad Espeleológica de Puerto Rico (SEPRI) and resurveyed by Ciudadanos del Karso (CDK) and is published here with their permission. The blue star corresponds to the air sampling done at the cave entrance and the orange star corresponds to the air sampling done inside the cave.


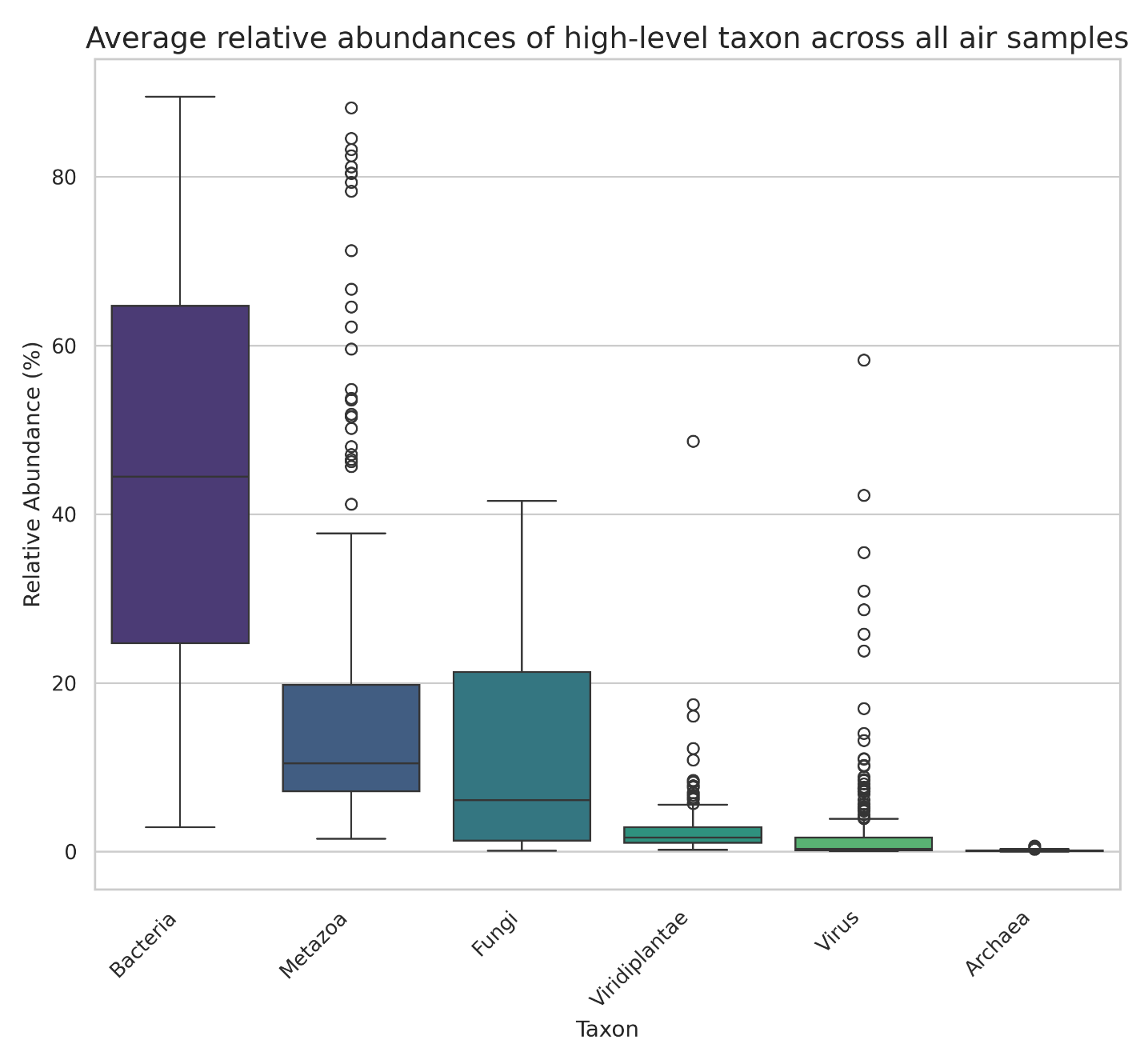


Supplemental Figure 2: High-taxon classification by Kraken2 shows the relative abundance of select domains and kingdoms across all air samples.
